# Immobilization of modular peptides on graphene cocktail for differentiation of human mesenchymal stem cells to hepatic-like cells

**DOI:** 10.3389/fchem.2022.943003

**Published:** 2022-08-29

**Authors:** Behzad Adibi-Motlagh, Ehsan Hashemi, Omid Akhavan, Jafar Khezri, Aram Rezaei, Javad Zamani Amir Zakria, Seyed Davar Siadat, Abbas Sahebghadam Lotfi, Abbas Farmany

**Affiliations:** ^1^ National Research Center for Transgenic Mouse, National Institute of Genetic Engineering and Biotechnology, Tehran, Iran; ^2^ Department of Clinical Biochemistry, Faculty of Medical Science, Tarbiat Modares University, Tehran, Iran; ^3^ Endocrinology and Metabolism Research Center, Endocrinology and Metabolism Clinical Sciences Institute, Tehran University of Medical Sciences, Tehran, Iran; ^4^ Department of Physics, Sharif University of Technology, Tehran, Iran; ^5^ Institute for Nanoscience and Nanotechnology, Sharif University of Technology, Tehran, Iran; ^6^ Nano Drug Delivery Research Center, Health Technology Institute, Kermanshah University of Medical Sciences, Kermanshah, Iran; ^7^ Department of Mycobacteriology and Pulmonary Research, Pasteur Institute of Iran, Tehran, Iran; ^8^ Dental Research Center, Hamadan University of Medical Sciences, Hamadan, Iran; ^9^ Dental Implant Research Center, Hamadan University of Medical Sciences, Hamadan, Iran

**Keywords:** graphene platform, differentiation, hepatic-like cells, *in silico*, modular peptide

## Abstract

In this study, two novel biomimetic modular peptide motifs based on the *alpha*-*2 subunit* of type *IV* collagen (CO4A2) were designed and immobilized on a graphene platform to imitate integrin and heparan sulfate- (HS-) binding proteins. The *in silico* study was used to design 9-mer K[KGDRGD]AG and 10-mer KK[SGDRGD]AG for testing designed Integrin-Binding Peptide (dIBP) and HS-Binding Peptide (dHBP). The virtual docking technique was used to optimize the peptide motifs and their relevant receptors. Molecular dynamic (MD) simulation was used to evaluate the stability of peptide-receptor complexes. The effect of the platform on the differentiation of human mesenchymal stem cells (hMSCs) to hepatic-like cells (HLCs) was evaluated. After differentiation, some hepatic cells’ molecular markers such as albumin, AFP, CK-18, and CK-19 were successfully followed. Graphene-heparan sulfate binding peptide (G-HSBP) enhances the mature hepatic markers’ expression instead of control (*p* ≤ 0.05). The pathological study showed that the designed platform is safe, and no adverse effects were seen till 21 days after implantation.

## Introduction

In advanced liver diseases, such as cirrhosis, many hepatocytes vanish, which cannot repair the liver tissue. Therefore, a liver transplant is required. However, because of the donor limitation and rejection problems, researchers urged to use cell therapy. In these patients, the lack of normal hepatocytes is a challenge. One choice is the differentiation of mesenchymal stem cells (MSCs) to hepatocyte-like cells (HLCs) on two-dimensional (2D) or three-dimensional (3D) biomimetic biomaterials (Jaiswal and Dhayal, 2020; Kraehenbuehl et al., 2011; Ramadas et al., 2017; Wang et al., 2020; Zujur et al., 2017). Using 2D or 3D microenvironments for differentiation purposes is preferable (Akhavan and Ghaderi, 2014; Lee et al., 2011; Suhito et al., 2017); however, it depends on the employed substrate bioactivation method (Asumda et al., 2018; Discher et al., 2009; Martino et al., 2009; Nayak et al., 2011; Kim et al., 2015; Stephanopoulos et al., 2015). Substrate bioactivation by ECM-derived proteins such as GAGs or HGF has disadvantages such as complex structural composition and immunogenic and labor working (Cai and Heilshorn, 2014; Graysona et al., 2009; Ghaedi et al., 2011; Lutolf and Hubbell, 2005). ECM-derived peptides as peptide motifs can mimic the function of ECM proteins with some benefits such as cheaper supplies, consistency, solubility, and exclusivity (Jhala and Vasita, 2015; Krishnamoorthy et al., 2018).

At this time, more than 250 peptide motifs are derived from ECM fibrous proteins. Most ECM-derived peptide motifs have been immobilized on different substrates, and their interactions with specific receptors have been studied. For example, GRGDSP, as a fibronectin-derived integrin binding peptide, activates α5β1 integrin (Defined three dimensional microenvironment for cell culture, 2018; Mota et al., 2013; Synthetically designed extracellular microenvironment, 2015). The most important issue with peptide immobilized biomaterials is their side effects and toxicity. Immobilized peptides cover the surface substrates preventing their interaction with cells and induce some essential mechanochemical signals for cell proliferation, development, and differentiation (Holle et al., 2008; Pan et al., 2016). Recently, MSC interaction with bioactive peptides immobilized on a graphene-based substrate is reported (Adibi-Motlagh et al., 2018). In this study, two novel biomimetic peptide motifs based on the *alpha*-*2 subunit* of type IV collagen (CO4A2) were designed and immobilized on a graphene platform to imitate integrin and heparan sulfate- (HS-) binding proteins for differentiation of MSCs to mature HLCs.

## Materials and methods

All experimental protocols were approved by the Ethics and Animal Handling Committee of the Hamadan University of Medical Science (IR.UMSHA.REC.1397.357). All methods were conducted in accordance with relevant guidelines and regulations.

### *In silico* study

6-mer peptide motif [GFPGER] and 7-mer peptide motif [TYRSRKY] were used as a positive control of Integrin-Binding Peptide (cIBP), and positive control of HS-Binding Peptide (cHBP), respectively (Hudalla and Murphy, 2010; Lee et al., 2007; Seo et al., 2010). Furthermore, two peptide motifs were selected from CO4A2, [KGDRGD] and [SGDRGD], which are used to design 9-mer K[KGDRGD]AG and 10-mer KK[SGDRGD]AG for testing Integrin-Binding Peptide (dIBP) and designed HS-Binding Peptide (dHBP).

### Three-dimensional structure predictions

PEP-FOLD3 was used to predict the 3D structure of peptides (Lamiable et al., 2016). 3D structure of HS (ID Code:53477714) was downloaded from PubChem structure, and the crystal structure of αvβ3 Integrin (ID Code:1JV2) was downloaded from PDB.

### Molecular docking study

Peptide motifs and their relevant receptors were optimized using Molegro Virtual Docker (MVD) version 4.0.2 (Thomsen and Christensen, 2006). Optimized structures were docked against peptides in 10 independent runs with the MolDock optimizer algorithm, and each run returned five poses. Poses obtained from 10 runs were ranked based on MolDock score, and the best score pose was selected as favorable peptide-receptor interaction. The simulation was repeated three times for each docking.

### Molecular dynamic simulation study

A molecular dynamic (MD) simulation study was used to evaluate the stability of peptide-receptor complexes using GROMACS4.5.5 and GROMOSE 54A7 as force fields to generate proper topologies (Hess et al., 2008). The peptide-receptor complexes were put in a cubic box and filled with water using the TIP3P model. Cl^−^ or Na^+^ ions were used as neutralizing agents. The steepest descent method was used for energy minimization. Periodic boundary conditions (PBC) were applied to avoid the edge effect interactions. All systems were equilibrated for 100 picoseconds (ps) under NVT and NPT at 300 K and 1 bar, respectively. 24,000 ps molecular dynamics simulation was run with no restraints. The root mean square plot (RMSD) shows the structural deviation of the peptide, which was plotted from the initial structure as a function of time. During the molecular dynamic simulation, peptide structure fluctuation was investigated by depicting root mean square fluctuation (RMSF). Binding free energy and the contribution of each residue in binding energy were calculated using the g_mmpbsa built-in function (available in GROMACS).

### Peptide immobilization

Details of graphene oxide (GO) synthesis are reported elsewhere (Adibi-Motlagh et al., 2018). Graphene film was prepared by chemical reduction of a mold-derived GO. Briefly, GO solution (15 mg/ml) was added drop-by-drop to a plastic mold (plate) with a 5 cm radius. The fabricated graphene film was subsequently treated by chemical reduction with HI (40%) for 24 h at room temperature. The film was washed carefully with deionized (DI) water to remove the residual acid and iodine and freeze-dried to fabricate the graphene film. Peptide immobilization on the graphene film was made according to previous studies (Adibi-Motlagh et al., 2018; Rezaei et al., 2016). In a typical experiment, a methanol solution of cyclohexyl isocyanide, an aqueous solution of formaldehyde (1:1 eq), and peptide solution (1.1 eq) were added to the graphene film surface in MES buffer at pH 6.1. The system was shaken for 30 min. The film was subsequently washed with 0.01 M MES buffer. The obtained film was eluted with a 0.5 M NaCl solution and stored at 4 °C.

### Cell culture, viability assay, and differentiation

5×10^5^ human mesenchymal stem cells (hMSCs) were cultured in a modified culture dish at a 5% CO_2_ incubator (37°C). MTT assay was used to evaluate the cell viability of the platform. Briefly, 1 × 10^4^ hMSCs were cultured in a 96-well plate, and after 24 and 48 h incubation times in a 5% CO_2_ incubator (37°C), the MTT solution was added to the wells. After 3 h of incubation, DMSO was added to the wells, and absorbance was read at 580 nm using an ELISA reader (Microplate reader labsystem multiscan). In order to differentiate hMSCs from HLCs, a two-step protocol was utilized. In the first step, early 7 days, cells were cultured in a 20 ng/ml HGF and 10^–7^ M DEX consisting of α-MEM supplemented with 10% FBS, and after cells attachment, in the 7th day, after the addition of inductive factors, 20 ng/ml HGF and 10^–7^ M DEX were followed for 2 weeks by 20 ng/ml OSM (Ghaedi et al., 2014).

### RNA isolation and quantitative (q) real-time PCR

Total RNA was extracted from treated cells using a high pure RNA isolation kit (Roche, Germany). The extracted RNA was stored at −80°C until use. cDNA was synthesized by the Fermentas kit (Thermo Fisher, United States) and stored at −20°C until use. The real-time PCR was carried out with an ABI System (Applied Biosystems StepOne, United States) under the following thermal conditions: 95°C for 2°min, 40 cycles of 95°C for 10 s, and 58°C for 30 s. Melting curves were analyzed after 40 cycles to confirm the PCR product specificity. Primer sequences used for qReal-Time PCR analysis are presented in Supplementary Table S1.

### Animal study

Fourteen-week adult Wistar rats were housed in controlled temperature and humidity conditions (20 ± 2°C on a 12:12 h light/dark cycle). Animals were shaved following anesthetization by ketamine/xylazine (100/12.5 mg/kg) procedure. The sterile designed platform was implanted subcutaneously between skin and fascia. The animal was closed by surgical suture and followed up for 3 weeks.

### Statistical analysis

All experiments were carried out in triplicate. The Shapiro–Wilk method was used to evaluate the data distribution. Student’s *t*-test and analysis of variance (ANOVA) were used to analyze experimental data. SPSS 21 software was utilized for analysis. *p* < *0.05* was considered statistically significant.

## Results and discussion

### In silico study

### Virtual docking

### Docking designed peptides with αvβ3 integrin and HS receptor

**FIGURE 1:**
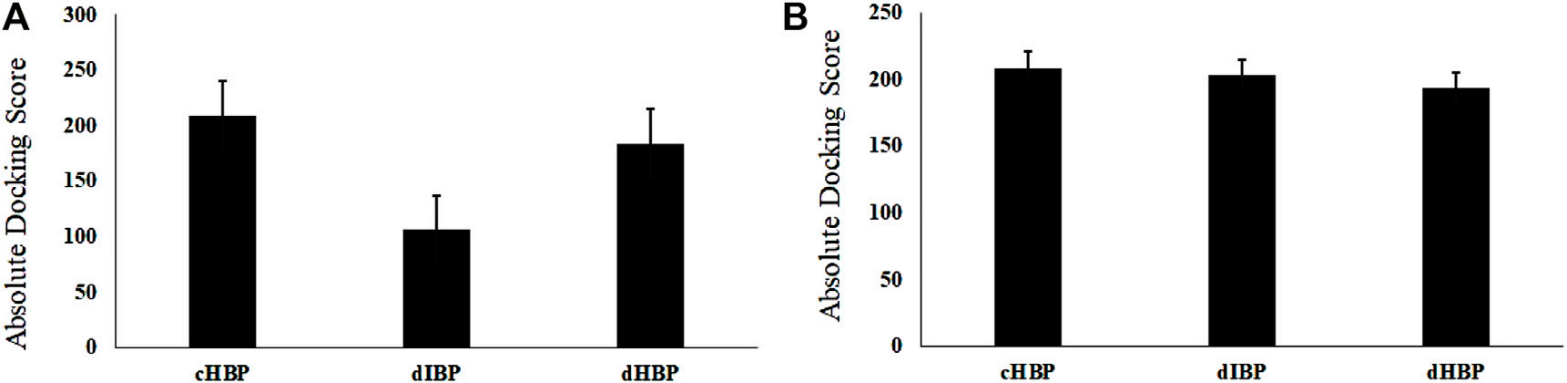
Graph of **(A)** integrin docking score; **(B)** HS docking score with designed peptides compared to positive control.

**FIGURE 2:**
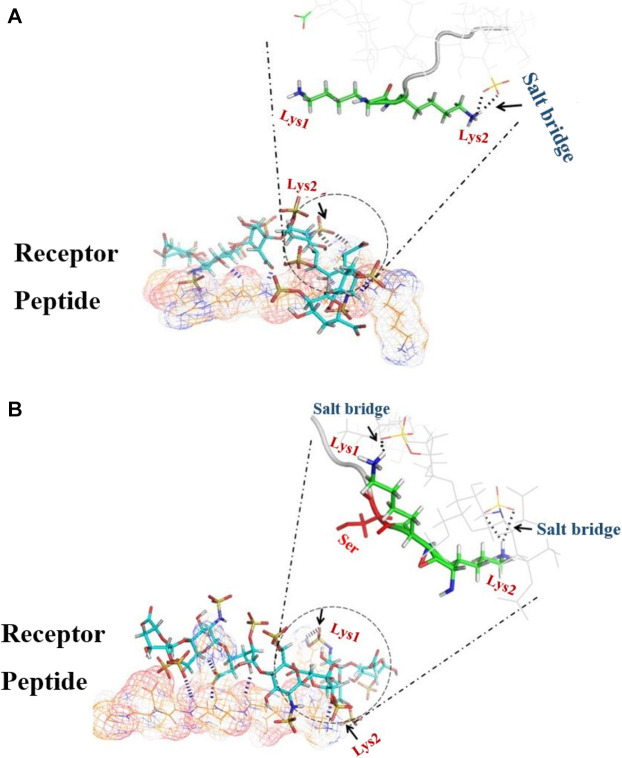
3D image of the dP-HS complex; the main interaction energy comes from hydrogen bonds (blue dashes) and salt bridges (black dashes). **(A)** In dIBP-HS, only Lys2 forms a salt bridge (black arrow) with HS. **(B)** In dHBP-HS, both Lys1 and Lys2 form a salt bridge with HS.

In this study, two novel CO4A2-derived peptide motifs, KGDRGD and SGDRGD, were designed. For this purpose, different peptides were docked against HS with three repeated runs. As shown in Figure 1A, the highest docking score is related to the peptides-integrin complex. However, dIBP and dHBP did not show a significant difference in binding to the receptor, and peptides exhibit the same behavior as cIBP-integrin docking. For adapting to a high affinity-repeat sequence and more flexibility, peptide motifs were modified to KK[SGDRGD]AG and K[KGDRGD]AG, which were designed as a ligand with high-affinity binding to HS, designed HS binding motif (dHBP) and binding to integrin receptor, designed integrin binding peptide motif (dIBP), respectively. Figure 1B shows the highest docking score of the peptides-HS complex. The control peptide motif (cHBP) binding to HS had no significant difference with the designed peptide (dHBP). Despite the similarity of high sequence to peptide, the peptide motif interaction with integrin receptor (dIBP) shows a lower affinity to HS (Figure 2).

Meanwhile**,** our bioinformatics studies complied with the results of Imberty et al., suggesting that cytokines through repeat sequence (KKXXXK) can bind to HS, but another repeat sequence (KKXXK) has less affinity to HS (Lortat-Jacob et al., 2002). A molecular docking study showed that the highest docking score with HS is related to *c*HSBP and followed by dHBP. Score docking of dIBP, despite the similarity to dHBP, shows the least susceptibility to HS. In fact, the presence of the third residue in Serine amino acid (^3^S) in the vicinity of lysine amino acid (^2^K) is a spatial position to form a salt bridge with HS (Figure 2A). In contrast, in the absence of residue ^3^S in dIBP, the Amine Lysine # 2 acid is not in a suitable position and cannot form a salt bridge with HS (Figure 2B). In fact, the presence of ^3^S in the vicinity of ^2^K approximated this residue to HS and salt bridge formation.

### Molecular dynamics simulation

Supplementary Figure S1 shows the RMSD of carbon alpha C^α^ for two peptides in complex with HS during simulation. Compared to dIBP, dHBP indicates lower RMSD, especially at the last frames (0.36 vs. 0.55). Therefore, C^α^ backbone peptide atoms in the dHBP-HS complex have a less deviation than the dIBP-HS complex over the last 24,000 ps. Therefore, there is no significant difference between docking results of designed peptides with integrin.

As shown in the plot, C^α^ atoms of dHBP peptides in complex with HS have less fluctuation during the simulation than dIBP, indicating more stability of the dHBP-HS complex (Supplementary Figure S1). Moreover, HS atoms are more rigid and stable in binding to dHBS (Supplementary Figure S1). In contrast, the total energy of dHBP-HS (blue) is twofold more than dIBP-HS (red in Supplementary Figure S1).

### Cell morphology and viability

**FIGURE 3:**
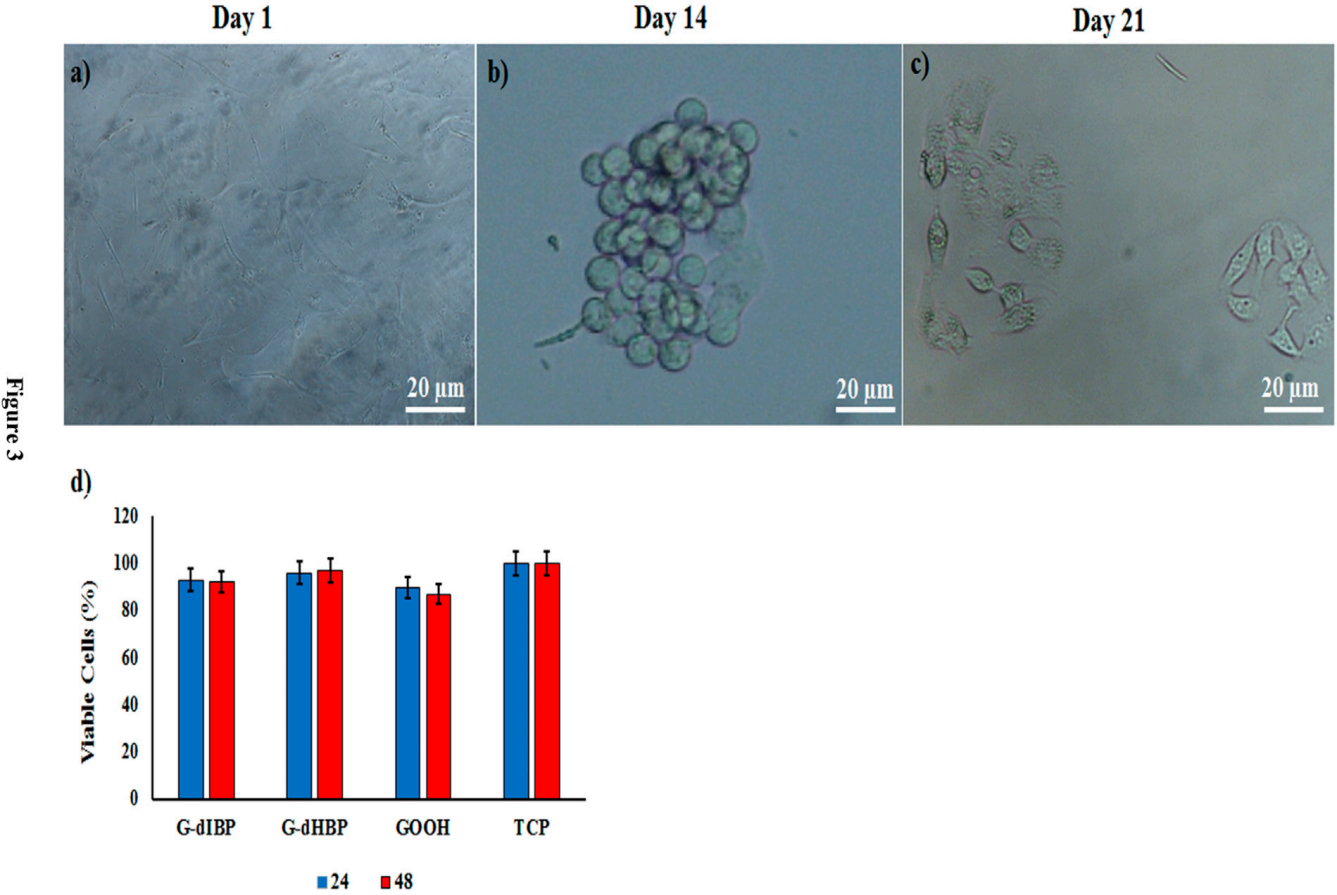
Time-dependent kinetics of MSCs differentiation to HLCs **(A,B,C)**. **(D)** Viability of cultured cells on different platforms. TCP indicates Tissue Culture Plate as control.

Morphological study of differentiated hepatic-like cells on the modified substrates shows that on the 7th day after the culture, cell differentiation was initiated. However, the microscopy images revealed that these changes were remarkable and clear on the 14th day after the culture; the differentiated cells were spherical and tended to make a clear colony (Figure 3B). After 21 days, the shape and morphology of cells were semi-HLCs. Cellular viability on G-dHBP, G-dIBP, and graphene platforms, after 24 and 48 h, were 96%, 97% and 93%, 92%, and 90% and 87%, respectively.

### CK18 and CK19 quantification

**FIGURE 4:**
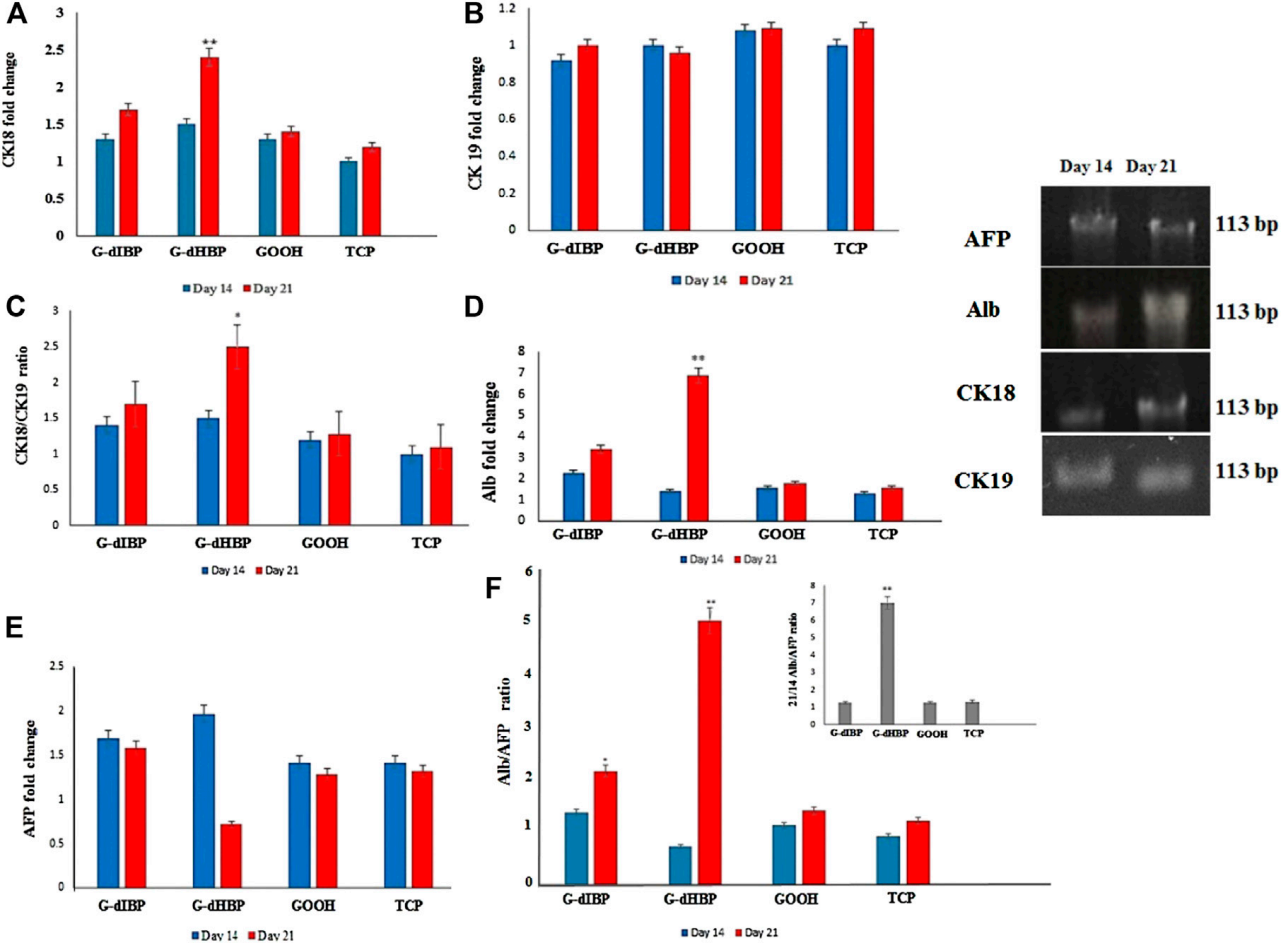
qRT-PCR of **(A)** CK18, **(B)** CK19, **(C)** CK18/CK19, **(D)** Alb, **(E)** AFP, and **(F)** Alb/AFP expression in different groups at 14 and 21 days after differentiation.

The CK18 and CK19 transcript levels were studied as the main hepatic cytoskeleton proteins. The obtained results showed that, on the 14th day after differentiation initiation, the CK18 transcript level was ∼1.4 for all groups, but it was greater in the G-dHBP platform (Figure 4A). As long as the differentiation time was 21 days after differentiation initiation, the transcript level reached 2.5-fold in the G-dHBP platform, which was statistically significant. However, the CK19 transcript level had no significant difference in different groups during the differentiation and even on the 21st day after differentiation initiation (Figure 4B). The mean quantitative gene expression of liver cytoskeleton proteins is presented in Figure 4. Comparison between CK18 and CK19 on the substrate containing dIBP, dHBP peptide, and controls was investigated on the 14 and 21 days after the cell culture (Figure 4). The mean CK18/CK19 ratio in differentiated cells on G-dHBP was higher than the rest of the groups, and on the 21st day after the culture, it was 2.5-fold. Furthermore, in the G-dIBP group, this ratio was greater than in controls (GOOH and TCP) (Figure 4C).

As the differentiation process has time-dependent kinetics, comparing the mature to immature marker ratios approves the process and validates the molecular dynamic simulation. Alb is a dominant protein produced by hepatocytes, which begins with initial embryonic hepatocytes and reaches the maximum in adult hepatocytes. AFP is a “fetal initial” marker, and its expression is reduced by liver development. Alb expression is an important marker of mature hepatocytes on the 21st day of differentiation, whereas AFP is a marker for immature hepatocytes. Fourteen days after the culture, the cell differentiation peaked, and AFP expression as an embryonic cell functional protein (immature) was turned-off instead of the functional protein of hepatocyte cells.

### Albumin and alpha-fetoprotein quantification

Quantitative real time-PCR was applied to obtain the marker genes transcript level in differentiated cells. Alpha-fetoprotein (AFP) is an embryonic factor mainly expressed in the early stage of cell development as long as cells mature. Quantitative real-time PCR results showed that the AFP transcript level was significantly decreased in the G-dHBP platform 21 days after the differentiation (*p* ≤ *0.05*) (Figure 4E).

In order to confirm the specificity effect of G-dHBP on hepatocyte-stage (maturity stage of HLCs), the cytoskeleton rearrangement markers expression (CK18/CK19) ratios were obtained (Figure 4C). Albumin (Alb) is a mature hepatic cell protein marker. The results of this study show that the Alb transcript level in differentiated cells in the G-dHBP platform (21 days after differentiation) was significantly greater (sevenfold) than that in other groups (*p* ≤ 0.01) (Figure 4D). The Alb/AFP expression ratio is obtained in all platforms. The results of this study show that the mean Alb/AFP expression ratio in differentiated cells at 14 and 21 days after differentiation is as follows: after 21 days of differentiation, the G-dHBP platform was significantly greater in all groups (*p* ≤ 0.001) (red dHBP column in Figure 4F). The ratio of 14 days after differentiation in the G-dHBP substrate was not significantly greater than that in other groups (Figure 4F). The Alb/AFP ratio was monitored at 14–21 days after differentiation (Figure 4F). The results show that at 21 days after differentiation, the Alb/AFP ratio was significantly greater in the G-dHBP platform (sevenfold) (*p* ≤ 0.001). However, the Alb/AFP ratio was not significantly different between G-dIBP, GOOH, and TCP (Figure 4F). The comparison between the ratio of HLCs mature to fetal marker (Alb/AFP) at 14 and 21 days after differentiation shows that this ratio reached its peak on day 21, whereas it was reversed on day 14 (Figure 4F). Briefly, the 21/14 Alb/AFP ratio increased sevenfold compared to controls (G-dIBP and GOOH) and TCP (Figure 4F).

### Platform

The graphene immobilized peptide synthesis and characterization were reported elsewhere (Adibi-Motlagh et al., 2018; Rezaei et al., 2016).

**FIGURE 5:**
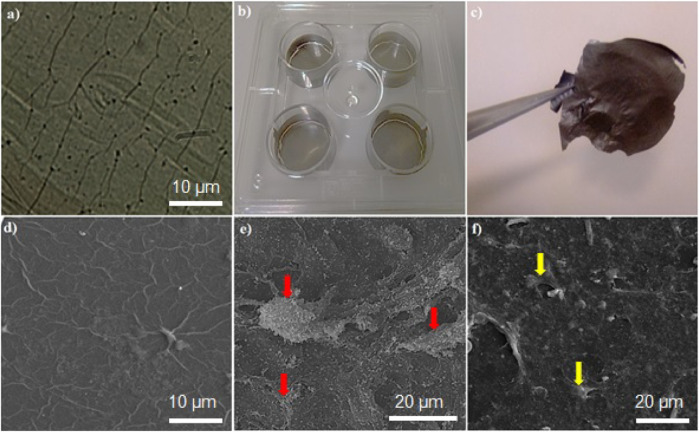
**(A)** Microscopic image of the graphene-based platform, **(B)** plate coated by graphene platform, **(C)** graphene film; SEM images of **(D)** the graphene-based platform, **(E)** hMSCs attached on graphene platform, **(F)** differentiated cells on the graphene-based platform. Red and yellow arrows show hMSCs and differentiated cells, respectively.

HMSC cells attached to the platform are shown in Figure 5E. As shown in Figure 5F, after 14 days of onset differentiation on the platform, the semi-hepatic phenotype cells were formed. Rolling up cells and their multi-face mode seems to be time-dependent. Cell morphological change is presented in Figures 5E,F. As shown in Figure 3, the mature cell nucleus and cytoplasm are rich in small vacuoles.

### Histopathology

**FIGURE 6:**
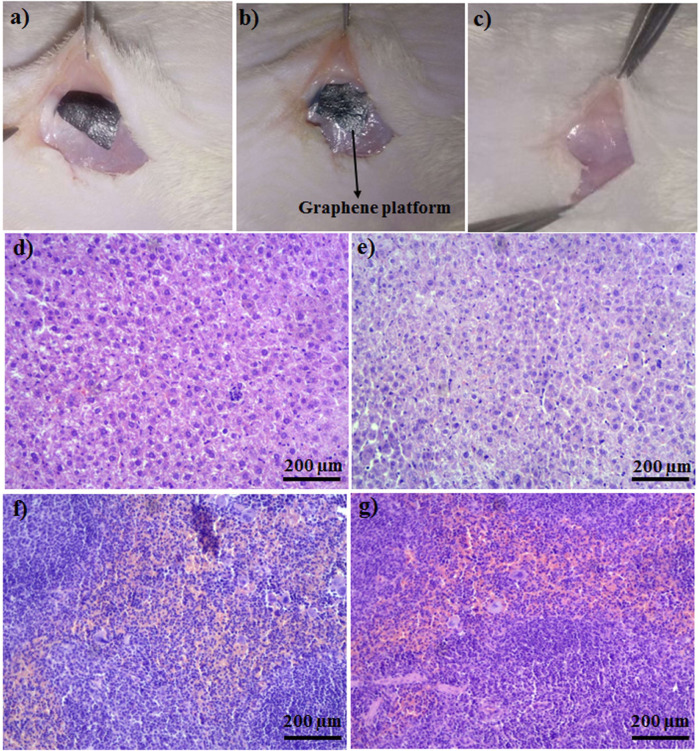
**(A)** Platform was implanted subcutaneously, **(B)** platform after 2 days of implantation, **(C)** control. Pathology images of **(D)** liver of control, **(E)** platform implanted liver, **(F)** spleen of control, **(G)** platform implanted spleen.

After 3 weeks of implantation of the graphene-based platform, the platform implanted in the livers and spleens of rats was checked (Figures 6A–C) and rats were followed up during this time period. During this time, no adverse effects, including feeding and drinking, or changes in animal weights during the treatment were observed. Histopathological results revealed no structural change in liver cells of treated rats with the implanted graphene-based platform. Veins, Kupfer, and hepatocyte cells were normal, and no remarkable differences between treated and non-treated samples were seen (Figures 6D,E). Normal architecture in both treated and control groups was clearly seen (Figures 6F,G, normal white and red pulps with any damages). The observation data and histopathological results revealed that the synthesized platforms are biocompatible in the *in vivo* system and have no side effects when it comes to *in vivo* applications.

## Conclusion

In conclusion, graphene platform and peptide motifs as physical and biochemical factors were used to make a biological cocktail for the regulation of *in vitro* cell fate. The biomimetic cocktail differentiated hMSCs to mature HLCs. The maturity of generated cells is mainly due to the use of designed peptide motifs that naturally manipulate the cell behavior. By applying the *in silico* approach, functional peptides were selected that, by making a biomimetic cocktail, successfully generated mature hepatic cells.

## Data Availability

The original contributions presented in the study are included in the article/Supplementary Material. Further inquiries can be directed to the corresponding authors.
