# Supplementary material for: Immobilization of modular peptides on graphene cocktail for differentiation of human mesenchymal stem cells to hepatic-like cells
Source: Front Chem. 2022 Aug 29;10:943003. doi: 10.3389/fchem.2022.943003 (PMC9465031; doi:10.3389/fchem.2022.943003)
Supplement: Supplementary file 1 [file DataSheet1.doc]

**Immobilization of Modular Peptides on Graphene Cocktail for Differentiation of Human Mesenchymal Stem Cells to Hepatic-Like Cells**

**Table S1. Primer sequences used for qReal-Time PCR analysis.**

| **Product size (bp)** | **Primer Sequence** | **Gene** |
| --- | --- | --- |
| **87** | F: 5ʹ TGCACCACCAACTGCTTAGC3ʹ  R: 5ʹ GGCATGGACTGTGGTCATGAG3ʹ | GAPDH |
| **114** | F: 5ʹ TGAGACGTACAGTCCAGTCCTT3ʹ  R: 5ʹ GCTCCATCTGTAGGGCGTAG3ʹ | CK18 |
| **103** | F: 5´TGAGTGACATGCGAAGCCAATAT3ʹ  R: 5ʹ GCGACCTCCCGGTTCAAT 3ʹ | CK19 |
| **70** | F: 5ʹ TTGCATGAGAAAACGCCAGTA 3ʹ  R: 5ʹ GTCGCCTGTTCACCAAGGA 3ʹ | Albumin |
| **131** | F: 5ʹ CTTTGGGCTGCTCGCTATGA3ʹ  R: 5ʹ GCATGTTGATTTAACAAGCTGCT3ʹ | AFP |


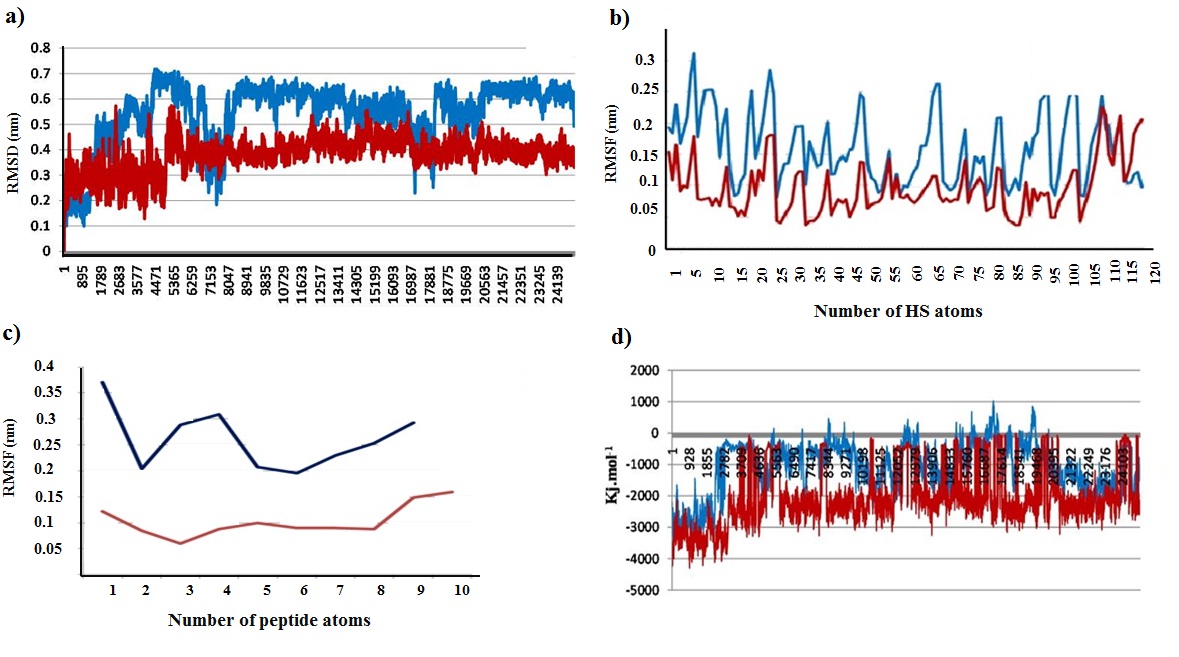


**Figure S1** a) The RMSD and b) RMSF diagram of the back bone of C^α^ peptide in complex of dHBP-HS (red graph), dIBP-HS (blue graph). c) The RMSF diagram of HS atoms in complex of dHBP-HS (red graph), dIBP-HS (blue graph). d) Total energy diagram of dIBP-HS complex (blue graph) is approximately more than 2 folds than dHBP-HS complex (red graph). X axis shows picoseconds.
